# The CanCURE Survey: Gender-Based Differences in HIV Cure Research Priorities

**DOI:** 10.3390/jpm15120623

**Published:** 2025-12-11

**Authors:** Jessica Lu, Branka Vulesevic, Shari Margolese, Renee Masching, Wangari Tharao, Claudette Cardinal, Tanguy Hedrich, Chris Mallais, Karine Dubé, Eric Cohen, Nicolas Chomont, Cecilia T. Costiniuk

**Affiliations:** 1 Faculty of Medicine and Health Sciences, McGill University, Montreal, QC H3G 2M1, Canada; jessica.lu3@mail.mcgill.ca (J.L.); renee.masching@outlook.com (R.M.); kokum6maskwa@gmail.com (C.C.); 2 Department of Medicine, Division of Infectious Diseases and Chronic Viral Illness Services, McGill University Health Centre, Montreal, QC H4A 3J1, Canada; 3 Canadian HIV Cure Enterprise (CanCURE) Community Advisory Board, Toronto, ON M4P 1G8, Canada; shari.margolese@gmail.com (S.M.); recherche@cocqsida.com (T.H.); chris.mallais@cdnaids.ca (C.M.); 4 Women’s Health in Women’s Hands Community Health Centre, Toronto, ON M5B 1J3, Canada; wangari@whiwh.com; 5 Division of Infectious Diseases and Global Public Health, University of California San Diego School of Medicine, La Jolla, CA 92093, USA; kdube@health.ucsd.edu; 6 Institut de Recherche Clinique de Montréal, Montreal, QC H2W 1R7, Canada; eric.cohen@ircm.qc.ca; 7 Département de Microbiologie, Infectiologie et Immunologie, Université de Montréal, Montreal, QC H2W 1R7, Canada; nicolas.chomont@umontreal.ca; 8 Centre de Recherche du Centre Hospitalier de l’Université de Montréal (CHUM), Montreal, QC H2X 0A9, Canada; 9 Infectious Diseases and Immunity in Global Health, Research Institute of McGill University Health Centre, Montreal, QC H4A 3J1, Canada

**Keywords:** HIV, HIV cure research, women with HIV, gender differences, research priorities, community engagement

## Abstract

**Background**: The Canadian HIV Cure Enterprise (CanCURE) is a pan-Canadian research collaboratory, investigating approaches for achieving sustainable HIV remission. In preparation for the next research cycle, CanCURE researchers and the Community Advisory Board (CAB) co-designed a web-based survey to identify HIV research priorities from the perspective of people with HIV (PWH) in Canada. The current study examined gender-based differences in these priorities. **Methods**: From August to December 2024, we recruited PWH across Canada through community organizations and community members. We collected data using REDCap electronic data capture tools hosted at The Research Institute of the McGill University Health Centre. The survey included 36 demographic questions, 16 questions related to general knowledge about HIV and HIV cure-related concepts, and 21 questions ranking research priorities. Knowledge questions were multiple choice, while priorities could be ranked on a scale. We summarized participant characteristics via descriptive statistics, and the research priorities were further stratified according to gender. **Results**: Of 109 participants, 48.6% self-identified as men, 46.8% as women, and 4.6% as two-spirit, non-binary, agender, or other. The median age was 53 years old. Approximately one-third of participants had lived with HIV for ≤14 years, one-third for 15–24 years, and one-third for ≥25 years. Overall, the median knowledge score of respondents was 79%. Among the 78 participants with prior HIV research experience, three times as many men (61.1%) as women (19.0%) participated in interventional studies involving medication or medical procedures. Men ranked preventing HIV transmission to partners as a priority, studying where the virus hides as the second, and avoiding high comorbidity risks as the third. In contrast, women ranked not having to take pills daily as a priority and avoiding higher risks for comorbidities as the second priority. Both genders equally valued expanding community involvement in HIV cure research. However, men focused more on integrating social and behavioural research, while women emphasized the need for diverse ethnic representation in research. **Conclusions**: Although both men and women share some common priorities regarding HIV cure research, there are notable gender differences in their specific concerns. Furthermore, a significant gender gap in participation in interventional studies, essential for advancing HIV cure research, highlights the importance of aligning research priorities with concerns of both genders.

## 1. Introduction

Since the advent of antiretroviral therapy (ART) in the 1990s, the management and epidemiology of HIV have undergone transformative changes [1,2,3]. By suppressing HIV viral load and maintaining adequate CD4+ T cell count, ART has significantly increased life expectancy and transformed HIV from a fatal disease into a chronic, but manageable condition. However, despite these advances, HIV remains incurable, and most people with HIV (PWH) must adhere to lifelong daily oral therapy. More recently, development of long-acting injectable ART, with some formulations possibly lasting up to six months, has introduced new possibilities and reshaped expectations for HIV care and cure strategies [4,5,6]. Amidst this evolving landscape of HIV management, the pursuit of an HIV cure has become a major scientific, community, and public health priority, encompassing biomedical, social, and ethical dimensions. Currently, two paradigms dominate HIV cure efforts: (1) a sterilizing cure, which aims to achieve a complete elimination of HIV from the body: similar to syphilis, malaria or tuberculosis, HIV can be completely cleared from the body (though re-infection is still possible); and (2) a functional cure, or durable ART-free control, in which HIV may still be in the body, but it is not active and cannot affect one’s health or be transmitted to others [7]. The definition of what constitutes a cure remains a subject of debate, with differing perspectives and priorities among scientists, clinicians, and community members [7,8,9,10]. A few rare cases have achieved an long-term immune control and HIV functional cure—where the virus remains undetectable without ongoing treatment—including the “Berlin” and “London patients” (after CCR5 Δ32 stem-cell transplants), the “Mississippi baby” (after ultra-early ART), and the “Barcelona patient” [11,12,13,14]. These exceptional instances highlight potential mechanisms for remission but remain rare, high-risk, and not yet generalizable to the wider HIV-positive population.

In 2022, an estimated 65,270 people were living with HIV in Canada, a quarter of whom were female [15]. In 2023, 2434 new HIV diagnoses were recorded. While sex refers to biological attributes, including hormonal fluctuations, anatomical distinctions, genetic factors, and varying responses to pharmacotherapy, gender encompasses socially constructed roles and behaviours [16]. Biological variables of sex have been shown to influence HIV reservoir and host immune response to cure strategies [7,17,18,19]. Social determinants of health, such as education, socio-economic status, gender-based violence and abuse, and stigma, interact with these biological factors, often exacerbating gender disparities in health outcomes [15,20]. Indeed, within the HIV cascade of care, women with HIV (WWH) are generally underdiagnosed and demonstrate lower adherence to care [21,22]. Factors contributing to this include social and structural inequities, such as unstable housing, financial constraints, gender-based violence, mental health issues (e.g., depression), and substance use disorders [23,24]. Additionally, pronounced racial disparities exist: whereas white men account for most men with HIV (MWH), Black and Indigenous women are disproportionately represented among WWH [18,19]. Despite these notable biological and social differences between sexes and genders, WWH remain consistently underrepresented in HIV cure studies, making up a median of only 11.1% of participants, according to a 2016 systematic review [19,25]. This underrepresentation limits the generalizability of scientific findings, potentially overlooking critical insights specific to WWH [7,26,27,28]. It is therefore essential to examine whether such disparities also extend to perceptions, priorities, and expectations surrounding HIV cure research to guide future research.

Although previous studies have explored the attitudes and expectations of PWH regarding a cure, none have been conducted in Canada or specifically examined gender-based differences in HIV cure research priorities. To address this gap, the Canadian HIV Cure Enterprise (CanCURE) and its Community Advisory Board (CAB) developed a survey to assess these priorities among PWH in Canada in preparation for the next funding cycle. This paper presents the survey findings and discusses their implications for future community-centred HIV cure research.

## 2. Materials and Methods

This project was led by the CanCURE 3.0 team, a pan-Canadian research consortium of basic scientists and CAB members investigating approaches for achieving sustainable HIV remission. This study had a cross-sectional design and consisted of a national online survey of PWH across Canada. The development of the survey involved the CanCURE team, various collaborators and CAB members, and took place in two stages. First, we performed a review of the literature, then we distributed a pilot survey to community members to receive and integrate their feedback before the general dissemination of the survey. Given the exploratory nature of the study, we aimed to achieve a sample size of 100, but no formal sample size calculation was conducted.

### 2.1. Development of the Survey

The survey was developed using previously published instruments, supplemented with new questions suggested by our Community Advisory Board (CAB). The initial draft was reviewed by the CAB to ensure clarity, ease of response, and appropriate use of sensitive language. Once the survey was programmed in REDCap, it was piloted with six participants who had previously collaborated with our team. One participant, a French-speaking lawyer, provided feedback on the French-language version, which was subsequently reviewed by francophone team members. The pilot participants suggested no major revisions to the survey content but recommended improvements to accessibility and the recruitment process, including increasing word-of-mouth outreach, highlighting the honorarium, and providing contact support for participants experiencing difficulties completing the survey.

### 2.2. Participants and Recruitment Strategy

CAB members and the CanCURE 3.0 team reached out to primary care providers and community organizations across the country, who referred participants to the CanCURE Survey coordination team. Community members also assisted in follow-up for incomplete responses. The coordination team shared the survey link with interested participants. Inclusion criteria were as follows: (1) HIV diagnosis; (2) 18 years of age or older; (3) referral from a physician or community organization; and (4) ability to complete an online survey in French or English. There were no exclusion criteria. To ensure a representative sample, we aimed to recruit a similar number of WWH and MWH as well as a diverse range of racial groups. Therefore, recruitment efforts were aimed at engaging various key demographic groups, such as WWH and individuals from racialized communities, through partnerships with CAB and community organizations that specifically support these populations (list can be found in Appendix A). Recruitment of participants took place over the span of 4 months, between August and December of 2024. Respondents received a $40 honorarium upon completion of the survey.

### 2.3. Survey Instrument

The questionnaire contained 36 demographic questions, which aimed to elucidate each participant’s demographic information, HIV management, prior participation in HIV research and knowledge about HIV, 16 knowledge questions on HIV and cure-related concepts, and 21 questions on cure research priorities. The full survey is available in Appendix B. Knowledge questions were multiple choice and were scored as correct, incorrect, or “not sure,” with participants also given the option to select “prefer not to answer.” Respondents evaluated HIV cure research priorities using two approaches: ranking and importance ratings. For ranking, participants were asked to place the listed priorities in a forced sequence, with “first” indicating the highest priority. In contrast, the importance ratings in the questionnaire were less restrictive, allowing participants to qualify each priority as “extremely important”, “important”, “neutral”, “very low importance” or “not at all important”. This dual approach allowed for the capture of both the relative ordering of priorities and the perceived significance of each, providing complementary insights.

Following informed consent, screened participants completed the online survey in REDCap, hosted on the Research Institute of the McGill University Health Centre’s web server. Only responses from completed surveys were included in the analysis.

### 2.4. Statistical Analysis

Given the exploratory nature of the study and the non-representative sampling approach, the analysis focused on identifying general patterns rather than testing specific hypotheses or generalizing to the broader population. Accordingly, analyses were limited to descriptive measures of the sample. Baseline characteristics are presented as frequencies and percentages. Knowledge scores and data on expectations, values and principles, and study participation are summarized both graphically and as percentages of respondents in each category.

### 2.5. Ethics Considerations

This study was conducted according to the Tri-Council Policy Statement Version 2 (TCPS2) and the principles in the Declaration of Helsinki. Ethics approval was obtained from the Research Ethics Boards (MUHC #2025-10776).

## 3. Results

### 3.1. Baseline Characteristics

In total, 109 PWH completed the survey between August and December 2024, of which 53 identified as men, 51 as women and 5 as two-spirit, non-binary, agender or other. Self-reported gender included cisgender and transgender individuals. Due to the limited number of gender-diverse participants, no meaningful trends could be derived from this sample. Fifty-six participants identified as White, 26 as Black, 9 as Latin American, 6 as Caribbean, 5 as South Asian, and 11 as Other. The median age of respondents was 53 years (IQR = 41–62). The duration of HIV diagnosis was ≤4 years in 8.3% of participants (n = 9/109), 5–9 years in 12.8% (n = 14/109), 10–14 years in 11.0% (n = 12/109), 15–24 years in 32.1% (n = 35/109), and ≥25 years in 35.8% (n = 39/109). Additional demographic information collected included assigned sex at birth, Indigenous identity, place of birth, highest level of education, student status, and employment status, as shown in Table 1. A sensitivity analysis of the baseline characteristics was conducted, confirming that variations in these characteristics did not meaningfully affect the results or alter the conclusions drawn from the data.

Of the 53 participants who identified as men, 35 were White, 7 were Black, 8 were Latin American, 3 were Caribbean, 3 were South Asian, and 2 preferred to describe their racial identity. Of the 51 respondents who identified as women, 18 were White, 19 were Black, 1 was Latin American, 3 were Caribbean, 2 were South Asian, 1 was Southeast Asian, 7 preferred to describe their racial identity, and 2 preferred not to answer. Of the 5 participants who identified as gender-diverse, 3 were White and 2 preferred to describe their racial identity (Table 2).

### 3.2. Knowledge About HIV and Cure-Related Concepts

The section of the survey on knowledge about HIV and cure-related concepts included multiple response formats. For checkbox questions, each statement about HIV cure-related research is treated as an independent item, with responses determined by a set logic prioritizing “Prefer not to answer,” checked items, “All of the above,” “None of the above,” and “Not sure.” Radio button questions allow a single response per item, with selections interpreted similarly: choosing a statement marks it as TRUE and others as FALSE, while “All of the above,” “None of the above,” and “Not sure” are handled consistently. Using this key for analysis, 19 correct answers were considered as 100% and so forward.

**Figure 1:**
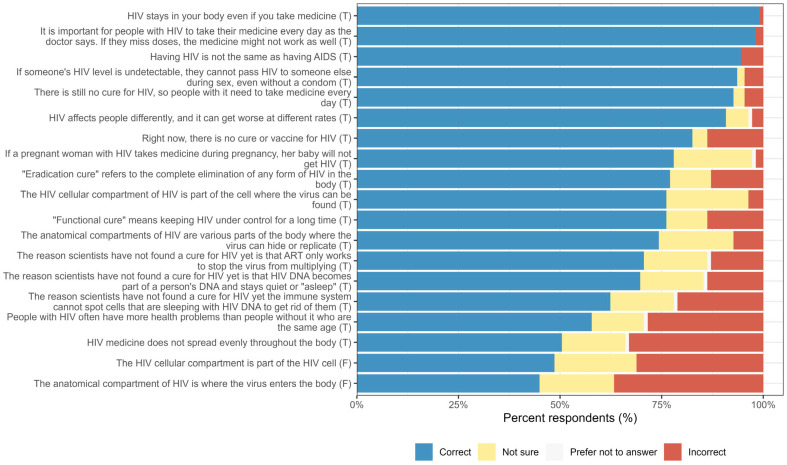
Percentage of respondents correctly, incorrectly, or uncertainly answering the HIV knowledge questions.

The median knowledge score of participants was 79% (IQR = 68–89%). As shown in Figure 1, most participants correctly answered questions about the persistence of HIV in the body despite treatment (99%, n = 108/109), the necessity of daily medication adherence (98%, n = 108/109), and the implications of ‘Undetectable equals Untransmittable’ (U = U) (94%, n = 102/109). However, knowledge was lower for questions about the differences between ‘sterilizing’ and ‘functional’ cures and the anatomical compartmentalization of HIV, with many respondents selecting ‘Not sure’ for these items. A significant proportion answered incorrectly regarding whether the HIV cellular compartment is part of the HIV cell (31%, n = 34/109) and whether the anatomical compartment is where the virus enters the body (37%, n = 40/109). Additionally, a notable fraction of respondents were uncertain or incorrect about the distribution of HIV medicine within the body (16%, n = 17/109 and 33%, n = 36/109, respectively).

**Figure 2:**
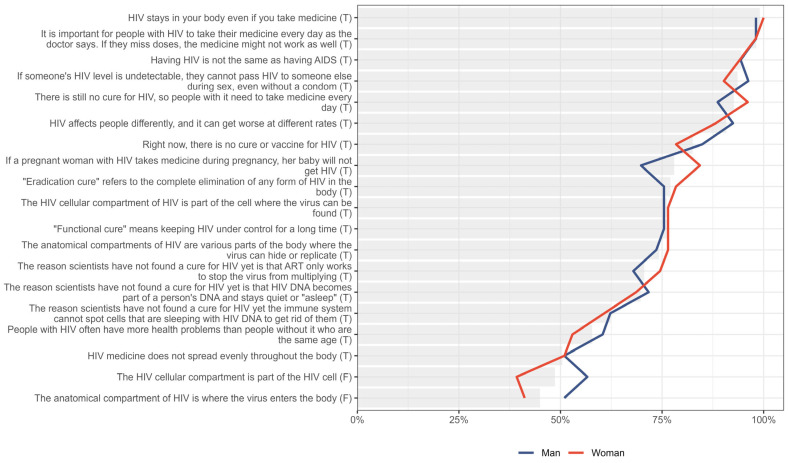
Percentage of participants correctly answering each knowledge prompt, stratified by gender. Gray bar indicated the overall percent for the entire sample.

Overall, knowledge scores between men and women were similar, as shown in Figure 2. The most notable difference lies in knowledge about HIV management during pregnancy: 84.3% (n = 43/51) of women correctly answered that if a pregnant woman with HIV takes medicine during pregnancy, her baby will not get HIV, compared to 69.8% (n = 37/53) of men. Additionally, a higher proportion of men than women correctly identified that ‘The HIV cellular compartment is part of the HIV cell.’ is a false statement (56.6%, n = 30/53 vs. 39.2%, n = 20/51).

**Figure 3:**
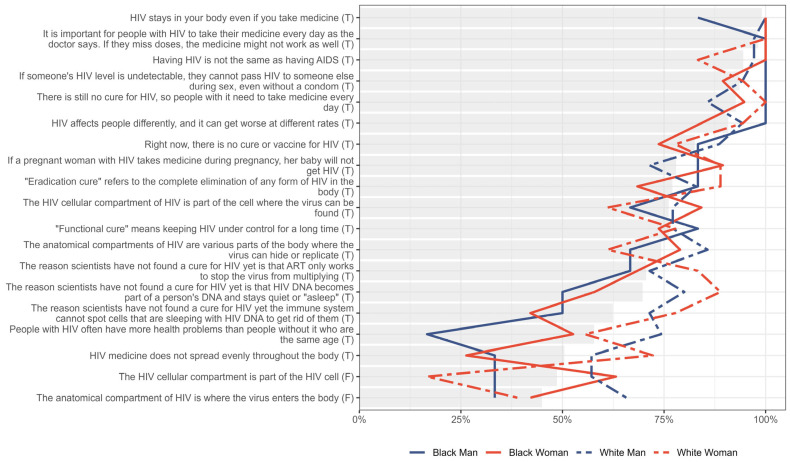
Percentage of participants correctly answering each knowledge prompt, stratified by gender and race. Gray bar indicated the overall percent for the entire sample.

Concerning knowledge levels stratified by gender and race, more stark discrepancies were identified in Figure 3. Across most items, White respondents, particularly White women, tended to have higher percentages of correct responses, while Black men had lower percentages on several items, notably those related to the distribution of HIV medication and detailed cure concepts. However, patterns were broadly similar across groups, with all groups showing lower knowledge on items regarding HIV cellular and anatomical compartments and sterilizing versus functional cures. While 16.7% (n = 1/6) of Black men correctly indicated that PWH often have more health problems than people without HIV who are the same age, 52.6% (n = 10/19) of Black women, 55.6% (n = 10/18) of White women, and 74.3% (n = 26/35) of White men knew that. Likewise, 50.0% (n = 3/6) of Black men, 57.9% (n = 11/19) of Black women, 80.0% (n = 28/35) of White men, and 88.9% (n = 16/18) of White women knew that HIV DNA becomes part of a person’s DNA and stays quiet or ‘asleep’ (HIV reservoir). Furthermore, 50.0% (n = 3/6) of Black men, 42.1% (n = 8/19) of Black women, 71.4% (n = 25/35) of White men, and 77.8% (n = 14/18) of White women correctly indicated that the immune system cannot spot cells with sleeping HIV DNA to get rid of them. Finally, 33.3% (n = 2/6) of Black men, 26.3% (n = 5/19) of Black Women, 57.1% (n = 20/35) of White men, and 72.2% (n = 13/18) of White women knew that HIV medicine does not spread evenly throughout the body.

### 3.3. Expectations

**Figure 4:**
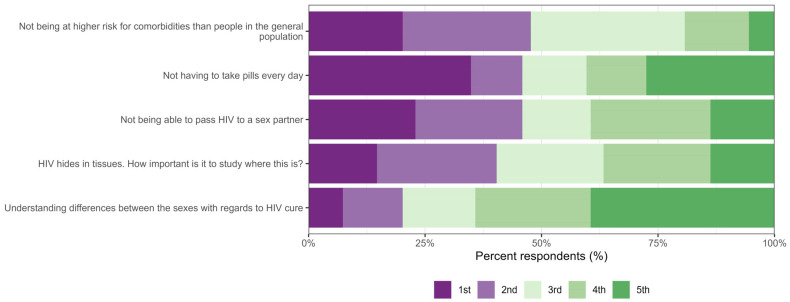
Distribution of priority rankings of five expectations in HIV cure research.

In the survey section where respondents ranked expectations for an HIV cure, Figure 4 shows the distribution of rankings across five key expectations. The most frequently selected top priority was ‘not having to take pills every day’ (35%, n = 38/109), followed by ‘not being able to pass HIV to a sex partner’ (23%, n = 25/109) and ‘not being at higher risk for comorbidities than people in the general population’ (20%, n = 22/109). In contrast, ‘understanding differences between the sexes concerning HIV cure’ was most often ranked as the lowest priority (5th) (39%, n = 43/109), with only 7% (n = 8/109) ranking it as their highest priority. The expectation to study HIV hiding in tissues received a broad range of rankings, mostly in the middle.

**Figure 5:**
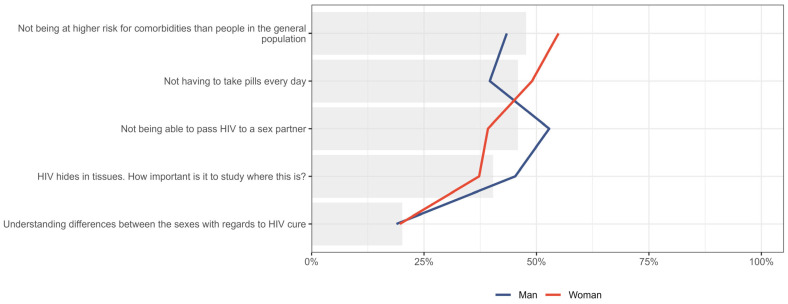
Distribution of participants who placed five expectations in HIV cure research as either first or second priority, stratified by gender. Gray bar indicated the overall percent for the entire sample.

When the first and second priority for HIV cure expectations were combined and analyzed by gender, notable differences were observed. The expectation of “not being able to pass HIV to a sex partner” was prioritized by 52.8% (n = 28/53) of men compared to 39.2% (n = 20/51) of women. In contrast, a higher proportion of women prioritized “not being at higher risk for comorbidities than people in the general population” (54.9%, n = 28/51, vs. 43.4%, n = 23/53) and “not having to take pills every day” (49.0%, n = 25/51 vs. 39.6%, n = 21/53) compared to men (Figure 5).

**Figure 6:**
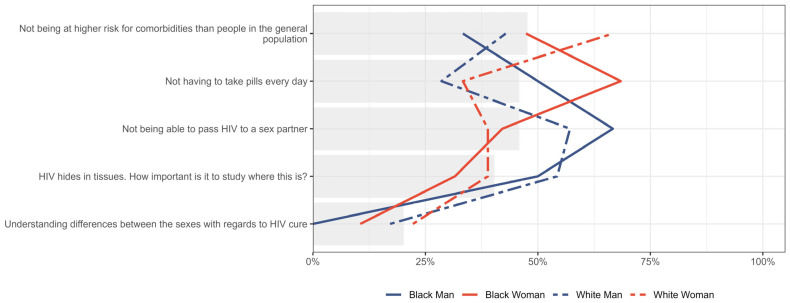
Distribution of participants who placed five expectations in HIV cure research as either first or second priority, stratified by gender and race. Gray bar indicated the overall percent for the entire sample.

When results were further stratified by gender and race, the patterns observed in the gender analysis remained prominent. Additionally, a higher proportion of Black men (50.0%, n = 3/6) and Black women (68.4%, n = 13/19) prioritized “not having to take pills every day”, compared to White men (28.6%, n = 10/35) and White women (33.3%, n = 6/18) (Figure 6).

### 3.4. Values and Principles

**Figure 7:**
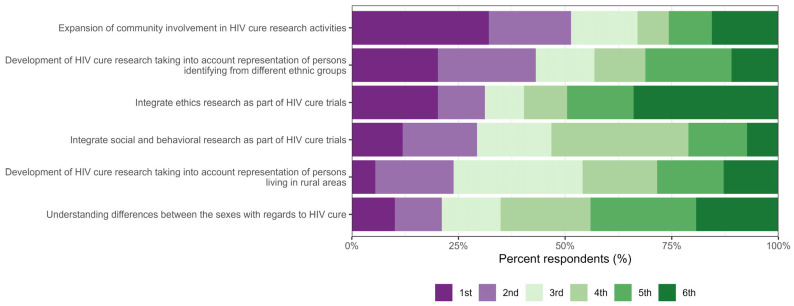
Distribution of rankings of six values and principles to be upheld in HIV cure research.

In a survey section asking respondents to rank a series of values and principles that researchers should uphold during HIV cure research, the “expansion of community involvement in HIV cure research activities” was most frequently ranked as the top priority (1st) (32%, n = 35/109), followed by “development of HIV cure research taking into account representation of persons identifying from different ethnic groups” (20%, n = 22/109) and “integration of ethics research as part of HIV cure trials” (20%, n = 22/109). In contrast, “integrating ethics research as part of HIV cure trials” was most frequently ranked lowest (34%, n = 37/109). Rankings for “taking into account representation of persons living in rural areas” and “integration of social and behavioural research as part of HIV cure trials” were more evenly distributed across middle ranks (Figure 7).

**Figure 8:**
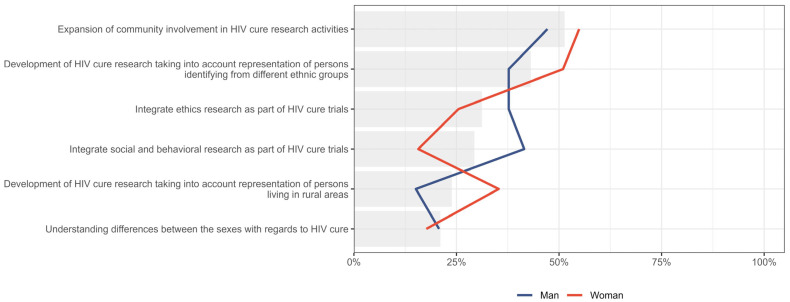
Distribution of participants who placed six values and principles to be upheld in HIV cure research as either first or second priority, stratified by gender. Gray bar indicated the overall percent for the entire sample.

The relative importance placed on different values and principles that researchers should uphold during HIV cure research, stratified by gender, is presented in Figure 8. Percentages indicate the proportion of participants within each group who ranked each value as their first or second priority. Both men and women prioritized the “expansion of community involvement in HIV cure research activities,” with 47.2% (n = 25/53) of men and 54.9% (n = 28/51) of women ranking it highly. Women placed greater emphasis on the “development of HIV cure research taking into account representation of persons identifying from different ethnic groups” (51.0%, n = 26/51, vs. 37.7%, n = 20/53 among men). In contrast, men prioritized “integration of ethics research as part of HIV cure trials” (37.7%, n = 20/53, vs. 25.5%, n = 13/51) and “integration of social and behavioural research” (41.5%, n = 22/53 vs. 15.7%, n = 8/51) more than women. Both genders accorded relatively less priority to “understanding differences between the sexes about HIV cure”.

**Figure 9:**
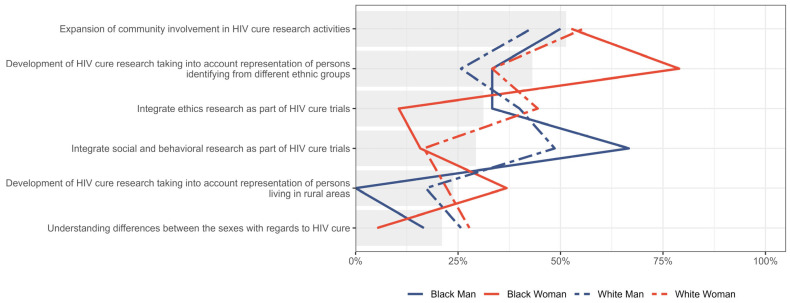
Distribution of participants who placed six values and principles to be upheld in HIV cure research as either first or second priority, stratified by gender and race. Gray bar indicated the overall percent for the entire sample.

Even stratified by gender and gender, the “expansion of community involvement in HIV cure research activities” was highly prioritized, with participants identifying as “Other” (58.1%, n = 18/31) and White women (55.6%, n = 10/18) according relatively the most importance, as shown in Figure 9. The most substantial gap is observed in Black women who attributed the most importance to the “development of HIV cure taking into account representation of persons identifying from different ethnic groups”, compared to Black men (33.3%; n = 2/6), White men (25.7%; n = 9/35) and White women (33.3%; n = 6/18).

### 3.5. Study Participation

**Figure 10:**
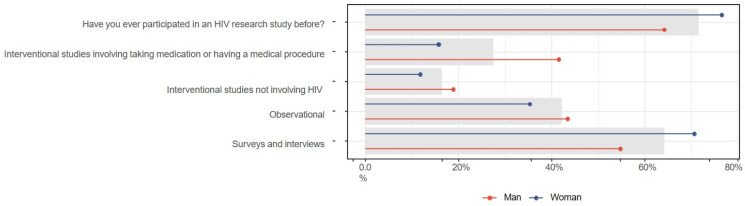
Prior participation in HIV research studies, stratified by gender. Gray bar indicated the overall percent for the entire sample.

The CanCURE survey included a section on respondents’ participation in HIV studies. Overall, 71.6% (n = 78/109) of respondents had previously participated in an HIV study; 64.2% (n = 34/53) of men indicated prior participation, compared to 76.5% (n = 39/51) of women, as shown in Figure 10. Nearly three times as many men as women had participated in interventional studies involving medication or medical procedures (41.5%, n = 22/53 vs. 15.7%, n = 8/51). A higher proportion of women than men in our study indicated participation in surveys and interviews (70.6%, n = 36/51 vs. 54.7%, n = 29/53), but slightly more men have been involved in observational studies (43.4%, n = 23/53, vs. 35.3%, n = 18/51).

**Figure 11:**
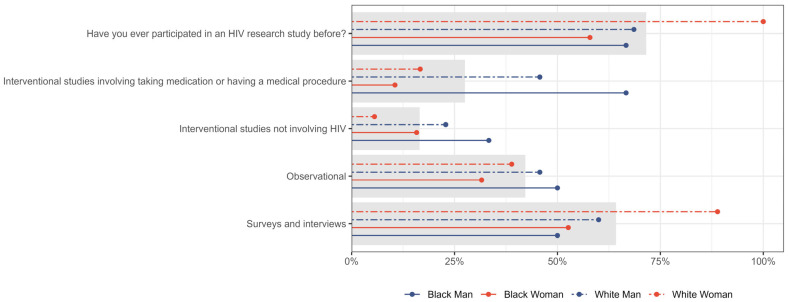
Prior participation in HIV research studies, stratified by gender and race. Gray bar indicated the overall percent for the entire sample.

Prior participation in HIV research studies was further stratified by gender and race in Figure 11. Participation rates in HIV research were highest among White women (100%; n = 18/18), compared to Black men (66.7%; n = 4/6) and White men (48.6%; n = 17/35), and Black women (57.9%; n = 11/19). Participation in interventional studies involving medication or medical procedures varied, with higher participation among Black men (66.7%, n = 4/6) and White men (45.7%, n = 16/35) in contrast to Black women (10.5%, n = 2/19) and White women (16.7%, n = 3/18).

### 3.6. Expectation for an HIV Cure Stratified by Knowledge About HIV and HIV Research

**Figure 12:**
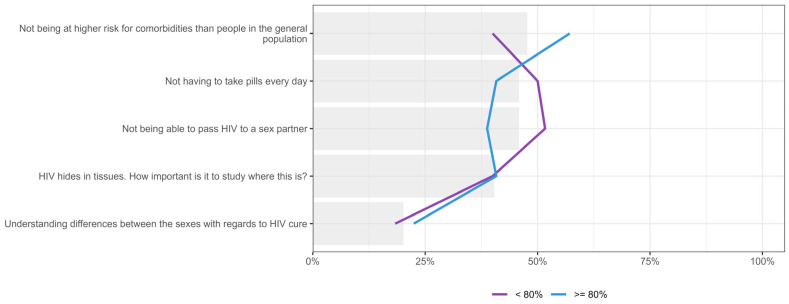
Distribution of participants who placed five expectations in HIV cure research as either first or second priority, stratified by knowledge (percent of correct answers). Gray bar indicated the overall percent for the entire sample.

We also analyzed the importance placed on different expectations for an HIV cure based on participants’ HIV knowledge levels (<80% vs. ≥80% correct on knowledge questions), as tested in the first part of the survey, by combining their first or second priority in Figure 12. Participants with higher HIV knowledge (≥80%) more frequently prioritized “not being at higher risk for comorbidities” (51.1%, n = 25/49 vs. 43.3%, n = 26/60) compared to those with lower knowledge levels. In contrast, those with lower HIV knowledge prioritized more highly “not being able to pass HIV to a sex partner” (51.7%, n = 31/60 vs. 38.8%, n = 19/49) and “not having to take pills every day” (50.0%, n = 30/60 vs. 40.8%, n = 20/49). Both groups showed similar prioritization for understanding HIV reservoirs, while “understanding differences between the sexes concerning HIV cure” was consistently ranked as a lower priority across both knowledge groups.

**Figure 13:**
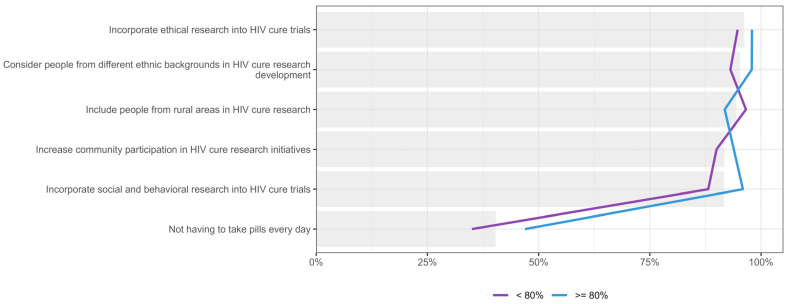
Distribution of participants who placed six values and principles to be upheld in HIV cure research as either first or second priority, stratified by knowledge. Gray bar indicated the overall percent for the entire sample.

When the importance placed on various values and principles for HIV cure research was stratified by participants’ HIV knowledge level, there was near-universal agreement on the importance of incorporating ethical research practices, considering people from different ethnic backgrounds, including people from rural areas, integrating social and behavioural research into HIV cure trials, and increasing community participation in HIV cure research initiatives, with endorsement rates above 91% for all categories, as show in Figure 13. In contrast, the importance of “not having to take pills every day” was endorsed less frequently, with 46.9% (n = 23/49) of participants with higher HIV knowledge prioritizing this expectation, compared to 35.0% (n = 21/60) among those with lower knowledge.

## 4. Discussion

Women are frequently underrepresented in HIV cure research studies compared to their male counterparts, thereby limiting the generalizability of HIV study findings [25,29,30]. WWH and MWH exhibit biological, immunological, anatomical, and hormonal differences that can influence the pathogenesis and the progression of HIV [31]. Likewise, gender-specific social determinants of health may vary tremendously, further exacerbating health outcomes [32]. To enhance the engagement of underrepresented PWH, particularly non-White and gender-diverse individuals, a nuanced understanding of their experiences is essential to address this lack of diversity in HIV cure research [33]. The rationale behind gender stratification in this study is thus to compare priorities between different populations, ensuring that future HIV cure strategies meet the needs of all PWH.

### 4.1. Knowledge About HIV and Cure-Related Concepts

Overall, respondents demonstrated high general knowledge about HIV treatment and transmission but showed lower accuracy and higher uncertainty regarding advanced concepts related to HIV reservoirs, cure definitions, and the anatomical and cellular compartmentalization of HIV, highlighting a knowledge gap in these areas. Interpretation of these findings should consider that many participants had prior involvement in HIV research (71.6%, n = 78/109), which may overestimate knowledge compared to the broader population of PWH. Most existing studies related to HIV knowledge have examined the understanding of HIV transmission, prevention, and treatment [34]. Few studies, however, have examined knowledge on HIV cure, likely due to the complex nature of the topic, making it challenging to situate our results within the literature [10,35]. While a limited understanding of advanced HIV cure concepts from respondents is not inherently concerning, it has important implications for trial readiness. Adequate comprehension of HIV cure-related concepts is essential to ensure informed decision-making and realistic expectations in HIV cure research. Thus, tailored, accessible educational strategies should be incorporated into community engagement and study protocols to support meaningful participation.

While knowledge scores were similar between men and women, a substantial discrepancy emerged in the domain of HIV management during pregnancy. Whereas 84.3% (n = 43/51) of women correctly indicated that if a pregnant woman with HIV takes medicine during pregnancy, her baby will not get HIV, only 69.8% (n = 37/53) of men were aware of this fact. This stark difference in our sample, if shown to be true in the broader population of PWH, may reveal the inherent influence of gender on health knowledge, particularly on topics related to reproductive health. The higher level of knowledge among women may reflect their closer adherence to reproductive health services, where HIV prevention strategies are often discussed. In contrast, men may have fewer links with healthcare services where such information is disseminated, highlighting a gap in male-focused health education regarding HIV transmission during pregnancy. This discrepancy underscores the potential need for tailored health education initiatives that engage men in discussions about HIV prevention in the context of family and reproductive health.

### 4.2. Expectations

The highest ranked expectation for an HIV cure for men in the study was to not be able to transmit HIV to a sex partner, echoing the findings of a global survey from 2017 in which 91% of PWH had indicated “no risk of passing HIV to sexual partners (even off treatment)” as a desired outcome for a potential HIV cure, despite the U = U paradigm being widely promoted [36]. The highest-ranked expectation for women was not to be at a higher risk for comorbidities than people in the general population, mirroring the findings of the previously mentioned study in which the most desired outcome for an HIV cure was “no risk of HIV-related health problems” [36]. Interestingly, in our study, only 52.9% (n = 27/51) of women knew that PWH often have more health problems than people without it who are the same age, compared to 60.4% (n = 32/53) of men. This disconnect between HIV knowledge and cure expectation potentially reflects the suboptimal communication about the long-term risks of HIV in clinical care.

The second highest expectation for men was to study where HIV hides in tissues. For women, it was not having to take pills every day, which echoes the pill burden experienced by many PWH [37]. In fact, half of PWH engaged in medical care take eight or more medications daily [38]. The literature extensively describes the challenges of consistently taking large pills, the stigma associated with visible medication use, and drug side effects; they may lead to suboptimal adherence or culturally purported cures, and cause emotional and physical distress in PWH [9,37,39]. The prospect of lifelong treatment fuels the strong community interest in an HIV cure that would remove the need for continuous drug adherence while reducing side effects and preventing onward transmission [36,37]. According to a meta-analysis from 2009, once-daily regimens of ART, more than twice-daily regimens, enhance adherence, thereby demonstrating the importance of addressing the concern of pill burden in HIV cure-related research [40]. Currently, women demonstrate poorer adherence to ART than men, and, in fact, female gender predicts lower access and adherence to ART, which may explain the high importance accorded by WWH in our study to not having to take daily pills [41].

### 4.3. Values and Principles

The highest-ranked value/principle for both women and men was the expansion of community involvement in HIV cure research activities, thus emphasizing the integral role of meaningful community engagement in shaping research priorities, ensuring ethical conduct, and improving recruitment and retention. As such, community members should be involved in all aspects of HIV cure study design.

While the second most highly ranked value/principle for men was the integration of social and behavioural research as part of HIV cure trials, for women, it was the development of HIV cure research considering the representation of persons identifying from different ethnic groups. This contrast may be due to the more ethnically diverse representation of women in our study, compared to men. In fact, our results show that Black women overwhelmingly placed more importance on this principle than Black men, White women, and White men. Overall, this result may suggest that WWH perceive existing research as insufficiently addressing the unique realities and barriers to care they face. Currently, few studies report on the representation of racial subgroups of WWH in HIV cure research, but the overall underrepresentation of WWH in HIV studies inherently limits the inclusion of diverse ethnic groups. In Canada, different ethnic groups of WWH face unique barriers to care that may hinder participation in HIV cure research because of suboptimal engagement in the health care system [42]. For example, compared to other racial subgroups of WWH, African, Caribbean, and Black women are more likely to experience involuntary disclosure of their HIV status and verbal and physical abuse, shaping both their relationship with their diagnosis and their interactions with the health care system. These challenges are compounded by interpersonal factors, such as trauma, fear and societal marginalization, all of which are reinforced by stigma rooted in systemic inequities [42]. Similarly, in the United States, 60.4% of Black WWH were found to have HIV-related medical mistrust, which adversely affects adherence to care [43,44]. Ensuring both gender and racial inclusivity in HIV cure research is therefore essential to build trust, reduce disparities in health outcomes, and enhance the validity of future studies.

### 4.4. Study Participation

Although a higher proportion of women than men in our study reported prior participation in HIV research, nearly three times as many men had participated in interventional studies involving medication or medical procedures. Since sex and gender are closely interrelated, the lack of women in interventional studies is especially concerning because they play a key role in advancing toward a cure by ascertaining HIV-related biological differences between sexes. This trend is consistent with existing literature: a 2015 review on the participation of females in global HIV cure research found that of 12,946 participants in studies with available sex data of participants, only 2323 (17.9%) were women, and less than 1% participated in cell therapy or reactivation studies, which are integral to HIV cure advancements [30]. While the overall enrollment of women in HIV cure trials (17.9%) roughly matches the sex distribution of the HIV population in Western and Central Europe, North America, and Australia, about one-quarter of HIV cure studies included no women at all [30]. The lack of representation of WWH may limit understanding of ART side effects in women, given the biological differences in HIV pathogenesis between males and females [30].

There are several possible reasons for women’s lower participation in interventional studies [45]. First, clinical trials involving HIV cure often require tissue sampling and blood collections to thoroughly study HIV reservoirs, making study visits long, frequent and complex. The collection of female-specific samples, particularly during female pelvic examination, may be a deterrent to potential participants. Second, although traditional recruitment strategies have been highly effective for engaging white men into studies, competing priorities for women, which include childcare and home responsibilities, often make these practices less successful. A lack of appropriate incentives, therefore, limits the engagement of diverse PWH. Third, a study on the participation of women in HIV clinical trials found that the most common reason for women’s non-participation was lack of information about research and not being offered enrollment, likely due to inadequate outreach efforts [7]. Fourth, inclusion criteria for women in HIV clinical trials can be conservative for pregnant women or women of childbearing age, further hindering female participation [25,46]. This caution may reflect gaps in knowledge about sex-specific responses to HIV treatment that cannot be resolved without including women in research, creating a self-perpetuating cycle. Fifth, women themselves may also have more medical mistrust than men, as we found in a study from our group, which examined reasons for vaccine hesitancy in PWH in Canada [47]. Women who distrust the healthcare system may be more reluctant to enroll in trials due to concerns about safety. Education and knowledge translation by trusted community members or elders may help address some of these barriers, especially for specific groups.

### 4.5. How to Guide Future Research

The literature consistently shows that the vast majority of PWH are eager to participate in HIV cure research, most often driven by altruistic motivations [36,48,49]. This willingness should be actively leveraged. However, the prospect of enrolling healthy PWH into trial studies without any expectation of a medical benefit and uncertain risks may raise ethical concerns. Ensuring an acceptable risk−benefit balance requires close consultation with HIV community groups and PWH to align HIV cure studies with participant needs, assess enrolment feasibility, and understand acceptable levels of risks of populations of interest [36,50]. Future studies should therefore strengthen close collaborations with community organizations supporting PWH during the study design process.

To ensure adequate gender representation, tailored enrolment of women is essential in all phases of clinical research. Although it may not be possible to recruit 50% women into every HIV cure study, a rate that matches population demographics must be a bare minimum. This may require changing recruitment strategies to targeted outreach efforts and appropriate incentives to overcome the unique barriers limiting women’s participation [7]. In HIV cure studies, sex-based analyses should also be conducted routinely to further elucidate biological differences in HIV pathogenesis, reservoir dynamics, and treatment response [7]. Similarly, given the emphasis WWH in our study placed on racial diversity in research, analyses stratified by race and sex or gender may be warranted to further understand intersectional factors affecting HIV outcomes.

### 4.6. Limitations

As this study is exploratory, it has several limitations. No formal sample size calculation was performed, so the results may not be representative of the broader PWH population. The sample size was insufficient to conduct adjusted analyses (e.g., logistic regression on top priorities) that could have strengthened the findings. Consequently, the results presented here should be interpreted as exploratory and descriptive, recognizing that a combination of gender and other sociodemographic factors may influence the observed unadjusted comparisons. The findings are intended to generate hypotheses rather than provide confirmatory evidence.

In this study, knowledge was assessed based on participants’ questionnaire scores; however, selecting the correct answer does not necessarily indicate true understanding. Additionally, the questionnaire did not include a dedicated section for soliciting concrete recommendations to guide future HIV cure research. Therefore, the suggestions presented in this paper are based on insights from both the survey responses and a review of the existing literature.

Most participants in our study have previously engaged in HIV research studies, creating a selection bias. In fact, prior study participation may indicate better health literacy and close engagement in the health care system, which does not necessarily reflect the reality of all PWH in Canada. Additionally, the provision of an honorarium may have influenced both who chose to participate and how they responded, potentially introducing further selection or response bias. Recruiting PWH who are less connected to healthcare or community networks is challenging due to factors such as stigma, mistrust, and limited access to communication channels. Future studies should establish targeted partnerships with LGBTQ+/Two-Spirit and other community organizations, utilizing diverse outreach methods to enhance the inclusion of underrepresented groups.

Additionally, we encountered challenges in recruiting gender-diverse participants, which limits the generalizability of our findings. Despite the high burden of HIV among transgender individuals globally, we did not conduct separate analyses for cisgender or transgender individuals [51]. Future studies investigating the main research priorities of gender minorities should therefore involve the collaboration of community organizations supporting these groups.

## 5. Conclusions

Overall, this study is the first in Canada to explore gender-based differences in HIV cure research priorities. Participants demonstrated higher knowledge levels about HIV treatment and transmission than about advanced HIV cure-related concepts. Both men and women share several common research priorities, but notable gender differences exist. For instance, more men prioritized the integration of social and behavioural research into clinical trials, and more women emphasized the importance of ensuring diverse racial representation in HIV cure studies. Furthermore, while most respondents indicated having participated in HIV studies in the past, more men than women participated in interventional studies, which are essential to the development of potential cures. Future cure studies must therefore address gender-specific concerns in research. Ultimately, aligning research priorities with community perspectives is critical, not only to enhance study participation but also to improve the relevance of findings across the diverse population of PWH. A broader survey on HIV cure priorities can identify the diverse values, concerns, and preferences of people with HIV, revealing heterogeneity in what outcomes matter most to different groups. By capturing these individual and subgroup perspectives, researchers can tailor study designs, communication strategies, and engagement approaches—moving beyond a one-size-fits-all model—to ensure that cure research is more relevant, acceptable, and impactful for the communities it aims to serve.

## Figures and Tables

**Table 1:** Demographic characteristics of participants.

Participant Characteristics	Value	n/N (%)
**Gender**	Man	53/109 (48.6%)
	Woman	51/109 (46.8%)
	Gender-diverse	5/109 (4.6%)
**Assigned sex at birth**	Male	54/109 (49.5%)
	Female	54/109 (49.5%)
	Other	1/109 (0.9%)
**Race**	White	56/109 (51.4%)
	Black	26/109 (23.9%)
	Latin American	9/109 (8.3%)
	Caribbean	6/109 (5.5%)
	South Asian	5/109 (4.6%)
	Arab	0/109 (0.0%)
	Filipino	0/109 (0.0%)
	West Asian	0/109 (0.0%)
	Self-descrribed	11/109 (10.1%)
	Prefer not to answer	2/109 (1.8%)
**Indigenous**	Yes	11/109 (10.1%)
	No	98/109 (89.9%)
**Place of birth**	Canada	63/109 (57.8%)
	Central Africa	11/109 (10.1%)
	South Africa	7/109 (6.4%)
	Europe	6/109 (5.5%)
	North America (not Canada)	6/109 (5.5%)
	Southeast Asia	5/109 (4.6%)
	Caribbean and Bermuda	4/109 (3.7%)
	Central America	3/109 (2.8%)
	North Africa	2/109 (1.8%)
	Eastern Asia	1/109 (0.9%)
	Prefer not to answer	1/109 (0.9%)
**Highest level of education**	Less than high school graduation	7/109 (6.4%)
	High school graduation	13/109 (11.9%)
	Non-university certificate or diploma from a community college, CEGEP	35/109 (32.1%)
	Trade certificate, vocational school, or apprenticeship training	12/109 (11.0%)
	University Bachelor’s degree	29/109 (26.6%)
	University graduate degree (Master’s, Doctorate, etc.)	12/109 (11.0%)
	Prefer not to answer	1/109 (0.9%)
**Student**	Yes	9/109 (8.3%)
	No	99/109 (90.8%)
	Prefer not to answer	1/109 (0.9%)
**Employment status**	Employed	50/109 (45.9%)
	Unemployed	48/109 (44.0%)
	Prefer not to answer	11/109 (10.1%)
**Work schedule**	Full-time	35/50 (70.0%)
	Part-time	15/50 (30.0%)
**Self-employed**	Yes	7/50 (14.0%)
	No	42/50 (84.0%)
	Prefer not to answer	1/50 (2.0%)
**On disability leave**	Yes	5/50 (10.0%)
	No	45/50 (90.0%)
**Stay-at-home parent**	Yes	7/48 (14.6%)
	No	40/48 (83.3%)
	Prefer not to answer	1/48 (2.1%)
**Retired**	Yes	28/109 (25.7%)
	No	78/109 (71.6%)
	Prefer not to answer	3/109 (2.8%)
**Caregiver**	Yes	14/109 (12.8%)
	No	92/109 (84.4%)
	Prefer not to answer	3/109 (2.8%)
**Length of HIV diagnosis**	4 years or less	9/109 (8.3%)
	5–9 years	14/109 (12.8%)
	10–14 years	12/109 (11.0%)
	15–24 years	35/109 (32.1%)
	25 years or more	39/109 (35.8%)
**HIV study participation**	Yes	78/109 (71.6%)
	No	19/109 (17.4%)
	Not sure	10/109 (9.2%)
	Prefer not to answer	2/109 (1.8%)
**Interventional (not HIV)**	Yes	18/78 (23.1%)
	No	52/78 (66.7%)
	Not Sure	8/78 (10.3%)
**Observational**	Yes	46/78 (59.0%)
	No	29/78 (37.2%)
	Not Sure	3/78 (3.8%)
**Survey or interview**	Yes	70/78 (89.7%)
	No	7/78 (9.0%)
	Not Sure	1/78 (1.3%)
**Knowledge score**	80% or higher	109/109 (100.0%)
	under 80%	0/109 (0.0%)

**Table 2:** Gender and race/ethnicity cross-tabulation.

	White	Black	Latin American	Caribbean	South Asian	Southeast Asian	Self-Identified	Prefer Not to Answer
**Man**	35	7	8	3	3	0	2	0
**Woman**	18	19	1	3	2	1	7	2
**Other**	3	0	0	0	0	0	2	0

## Data Availability

Deidentified data are available from the corresponding author upon reasonable request, subject to a data sharing agreement.

## Abbreviations

The following abbreviations are used in this manuscript:

CanCURE	Canadian HIV Cure Enterprise
CAB	Community Advisory Board
PWH	People with HIV
ART	Antiretroviral Therapy
WWH	Women with HIV
MWH	Men with HIV

## Appendix A

List of All Collaborating Community Organizations

AIDS New Brunswick (New Brunswick)

Alliance of South Asian AIDS Prevention (Ontario)

Canadian AIDS Society

Canadian Positive People Network

CAYR (Ontario)

Coalition des organismes communautraires québécois de lutte contre le SIDA (Québec)

Gay Men’s Sexual Health Alliance (Ontario)

HIV Edmonton (Alberta)

PAN (British Columbia)

Positive Leadership Development Institute (Ontario, Québec, British Columbia)

Regional HIV/AIDS Connection (Ontario)

Women’s Health In Women’s Hands (Ontario)

## Appendix B

Full Questionnaire on HIV Knowledge and Research Priorities of People with HIV
